# A systematic review of the cost and cost-effectiveness of immunoglobulin treatment in patients with hematological malignancies

**DOI:** 10.1017/S026646232400028X

**Published:** 2024-05-16

**Authors:** Sara Carrillo de Albornoz, Khai Li Chai, Alisa M. Higgins, Dennis Petrie, Erica M. Wood, Zoe K. McQuilten

**Affiliations:** 1 School of Public Health and Preventive Medicine, Monash University, Clayton, VIC, Australia; 2 Centre for Health Economics, Monash Business School, Monash University, Clayton, VIC, Australia

**Keywords:** hypogammaglobulinemia, immunoglobulin, hematological malignancies, cost, cost-effectiveness

## Abstract

**Objectives:**

Patients with hematological malignancies are likely to develop hypogammaglobulinemia. Immunoglobulin (Ig) is commonly given to prevent infections, but its overall costs and cost-effectiveness are unknown.

**Methods:**

A systematic review was conducted following the PRISMA guidelines to assess the evidence on the costs and cost-effectiveness of Ig, administered intravenously (IVIg) or subcutaneously (SCIg), in adults with hematological malignancies.

**Results:**

Six studies met the inclusion criteria, and only two economic evaluations were identified; one cost-utility analysis (CUA) of IVIg versus no Ig, and another comparing IVIg with SCIg. The quality of the evidence was low. Compared to no treatment, Ig reduced hospitalization rates. One study reported no significant change in hospitalizations following a program to reduce IVIg use, and an observational study comparing IVIg with SCIg suggested that there were more hospitalizations with SCIg but lower overall costs per patient. The CUA comparing IVIg versus no Ig suggested that IVIg treatment was not cost-effective, and the other CUA comparing IVIg to SCIg found that home-based SCIg was more cost-effective than IVIg, but both studies had serious limitations.

**Conclusions:**

Our review highlighted key gaps in the literature: the cost-effectiveness of Ig in patients with hematological malignancies is very uncertain. Despite increasing Ig use worldwide, there are limited data regarding the total direct and indirect costs of treatment, and the optimal use of Ig and downstream implications for healthcare resource use and costs remain unclear. Given the paucity of evidence on the costs and cost-effectiveness of Ig treatment in this population, further health economic research is warranted.

## Introduction

People with hematological malignancies are at higher risk of infections due to underlying immune deficiencies and treatment-related immunosuppression. Acquired hypogammaglobulinemia (HGG) is common in this population, and prophylactic immunoglobulin (Ig) is usually given to prevent and manage infections (1). Therapeutic innovations, such as B-cell targeted therapies and monoclonal antibodies, have led to improved survival but increased the incidence of HGG in patients with hematological malignancies (1–3). Previous systematic reviews have reported that Ig replacement therapy reduces infections in patients with hematological malignancies, but the quality of the evidence was considered low, the number of participants was small, and the majority of included trials were published before 2000 (4–6). Other interventions used to prevent infections in patients with hematological malignancies include vaccinations and prophylactic antibiotics. A systematic review by Chai et al. (5) reported that only prophylactic Ig and vaccinations reduced the risk of clinically documented infections, although the authors highlighted the high risk of bias in the studies.

Ig products are fractionated from human plasma through a complex and costly process (7). Ig use is the most important driver of plasma collection, contributing to the global imbalance between plasma collection and demand for plasma-derived medicinal products (8). Plasma supply in most European countries comes from unpaid plasma donations, and approximately 60 percent of plasma is imported from US remunerated donations (8;9). This increases the risk of Ig shortages, which have occurred over the past decade and during the COVID-19 pandemic due to reductions in plasma collection and disruptions in supply chains (10–12). A number of national authorities have developed Ig shortage management plans that prioritize patients at the highest risk (9;11–13). N’kaoua et al. (11) examined the impact of Ig shortages on patients with neurological conditions; 78 percent had Ig treatment modifications and 52 percent experienced clinical deterioration. The implications of shortages for patients with hematological malignancies remain unclear.

Annual demand for Ig has risen by 6 to 11 percent worldwide (7;14;15), generating a high economic burden for health systems. There are multiple clinical conditions competing for Ig treatment. A recent review (9) suggested that indications for Ig use have not changed considerably over time and therefore the increase in Ig demand may be due to more patients being diagnosed with currently approved indications, the administration of larger amounts of Ig per patient, and Ig use for indications unsupported by evidence.

An analysis of Ig reimbursement data in Belgian hospitals calculated a total annual Ig product cost of €33.5 million across approved conditions and off-label indications in 9,629 patients, which accounted for 17 percent of total hospital drug expenditure. The Ig treatment of 1,494 patients with secondary immunodeficiency or bone marrow transplantation amounted to €4 million (16). In France, the annual mean cost of Ig treatment per patient with secondary immunodeficiency has been estimated at over €20,000, of which €9,800 were Ig product costs and the remainder were hospital admission costs for Ig infusions and infections (17). In Australia, Ig product costs account for 50 percent of the total national budget for blood products, and HGG following hematological malignancies and/or hematopoietic stem cell transplant (HSCT) is the indication where the greatest amount of Ig is issued (15). It has been hypothesized that the cost of Ig treatment in this population might be offset by a reduction in antibiotic use, infection-related hospitalization days, and loss of working days (4;6), but there is no evidence to support this hypothesis, and the full cost of Ig treatment and cost-effectiveness in hematological malignancies remain unknown.

This aim of this review was to assess the health economic evidence for Ig treatment in order to better understand associated costs, healthcare resource utilization, and cost-effectiveness in patients with hematological malignancies.

## Materials and methods

This systematic review was designed following the PRISMA 2020 updated guidelines (18). The protocol was prospectively registered on PROSPERO (CRD42022321908).

### Search methods and selection criteria

The eligibility criteria followed the PICOS framework. We included studies published in English with a population of adult patients (≥18 years) with hematological malignancies treated with Ig, administered either intravenously (IVIg) or subcutaneously (SCIg). Comparators included no Ig therapy, other Ig administration routes (IVIg or SCIg), or no comparator. Studies that reported cost-effectiveness outcomes, health system costs, and resource utilization associated with Ig treatment were considered. Given the limited economic data in this therapeutic area, all study designs were included except reviews, case reports, commentaries, and editorials. Conference abstracts were excluded due to the inability to assess their methodologies. Nevertheless, relevant abstracts were reviewed to identify subsequent peer-reviewed publications.

The following databases were searched on March 29, 2022: Medline, EMBASE, Cochrane Central Register of Controlled Trials, Cochrane Database of Systematic Reviews, National Health Services Economic Evaluation Database, Database of Abstracts of Reviews of Effects, and Health Technology Assessment. A bibliographic search of systematic reviews and gray literature was also conducted.

The search strategy combined medical subject headings and key words specific to Ig treatment and hematological malignancies (e.g., lymphoma, multiple myeloma, chronic lymphocytic leukemia [CLL]). A number of economic terms were incorporated to identify economic evaluation and costing studies. The search was limited to the English language but was not restricted by date. The full search strategy is provided in the Supplementary material (Supplementary Table S1). The searches were updated while the manuscript was undergoing peer review, on December 6, 2023, and screened by a single reviewer (601 citations with no relevant studies found).

### Data collection and analysis

Two reviewers (S.C.d.A. and K.L.C.) independently assessed the retrieved citations in two steps: first, title and abstracts were assessed against the predefined eligibility criteria and irrelevant citations were excluded; second, full-text publications that met the inclusion criteria in the first step were reviewed and reasons for exclusions were recorded on a spreadsheet. Disagreements were resolved by a third reviewer (A.M.H.).

The following data were extracted by two reviewers (S.C.d.A. and K.L.C.) independently using a standardized Excel sheet: first author and date, country, design, and duration of the study, country, patient population, Ig type and dosing, attrition, and key outcomes. Discrepancies were resolved through discussion or adjudication by a third reviewer.

Two authors (S.C.d.A. and K.L.C.) independently assessed the quality of the included studies, and any discrepancies were resolved by discussion or a third reviewer (A.M.H.). The wider eligibility criteria with respect to study design resulted in the inclusion of a variety of study designs reporting economic and resource use outcomes. There is currently no quality checklist validated for use across study types and designs; therefore, different instruments were used for different study designs. The Cochrane risk of bias tools RoB2 and ROBINS-I were used to assess bias in randomized controlled trials (RCTs) and non-randomized studies, respectively (19;20). Several checklists are currently available to assess the reporting quality and applicability of economic evaluations, but no individual checklist has been recommended as the gold standard (21). We chose the most recently updated Consolidated Health Economic Reporting Standards (CHEERS) 2022 (22) to assess the quality of reporting of the economic evaluations, which has been proposed for the appraisal of health economic evaluations by The National Institute for Health and Care Excellence (23) in the United Kingdom.

A narrative synthesis of the evidence was conducted, given the paucity of data and high level of heterogeneity across the studies.

## Results

**Figure 1.**
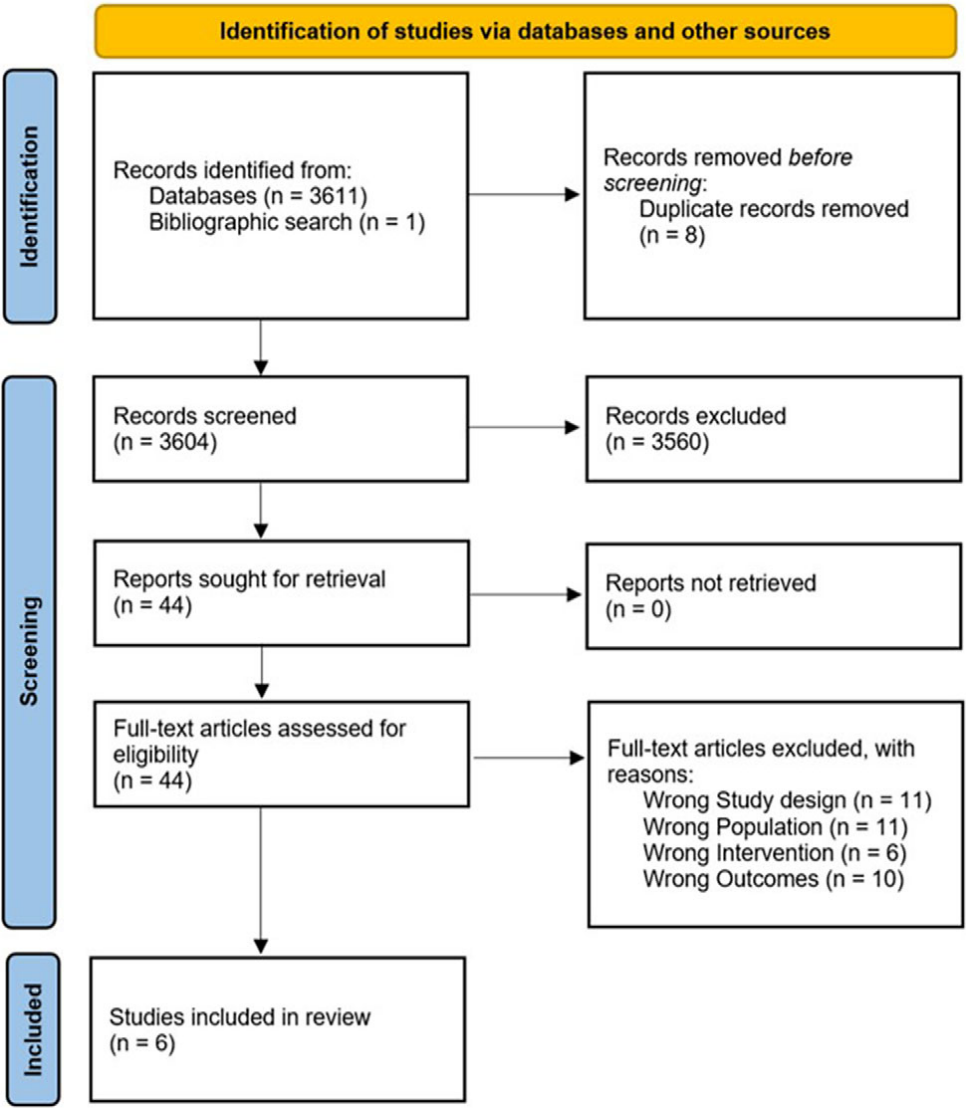
Flow diagram of the study selection process.

A total of 3612 citations were identified (Figure 1). Following the removal of duplicate records and title and abstract screening, 44 full text articles were assessed for eligibility and reasons for exclusion noted, and 6 studies were included in this review.

There was a high level of heterogeneity across the included studies, with different study designs, populations, comparisons, and outcomes (Table 1). Of the six studies that met our inclusion criteria, only two were economic evaluations of Ig. The remainder included one RCT and three observational studies that reported hospitalizations or costs alongside the primary outcome of infection incidence. Patient populations were mostly comprised of patients with HGG and CLL or MM, but varied across the studies. The severity of HGG differed across included studies, with different definitions of HGG, or IgG threshold (which indicates HGG severity) unspecified. The comparisons included SCIg or IVIg versus no Ig, IVIg versus reduced use of IVIg, and IVIg versus SCIg. Ig dosing and intervals varied across the studies; most dosing schedules comprised IVIg given at 0.4 g/kg every 3 to 4 weeks or SCIg weekly at 0.1 g/kg; two of the studies used reduced dosage or treatment intervals; and the RCT used a monthly SCIg dose ranging from 0.4 to 0.8 g/kg divided into weekly infusions with frequency adjusted according to IgG levels. Two studies were published before 2000 and four between 2018 and 2020. Most studies had very small patient numbers, and the quality of the evidence was poor. In particular, the observational studies were at serious risk of bias due to the selection of participants and confounding. Details of the quality assessment are provided in the Supplementary materials (Supplementary Table S2).

**Table 1.** Summary characteristics of included studies

References	Study design	Country	Population	Interventions	Study duration	Relevant outcomes
Derman et al. (24)	Observational study, before/after IVIg Stewardship program	USA	HM (CLL, MM, post–CAR T–cell, post–HSCT) and HGG (IgG <400 mg/dL). *N* = 274 Before program: IVIg/summer *n* = 86 IVIg/autumn, *n* = 48 After program: IVIg/autumn, *n* = 55 No IVIg/autumn *n* = 47	Before/after IVIg stewardship program as follows^:^ CLL/MM: treatment interval 6 weeks, IVIg (0.4 g/kg) administered if IgG <400 mg/dL AND ≥ 1 suspected or confirmed severe bacterial infection within the last 3 months that required (1) antibiotics and (2) hospital admission or ED visit After HSCT/CAR–T: treatment interval 3 to 4 weeks, IVIg (0.4 g/kg) administered if IgG <400 mg/dl at least 100 days after HCT or CAR–T (IVIg details before program not reported)	9 months: 3 months before and 3 months after. Plus a separate autumn pre–program subgroup (extra 3 months)	ED or inpatient admission due to infection (%), deaths from infection
Jurlander et al. (25)	Observational study, before/after IVIg	Denmark	CLL, HGG (IgG threshold undefined) and a history of recurrent infections. *N* = 15	Before IVIg/after fixed low dose IVIg (10 g every 3 weeks)	24 months: (12 months before and 12 months after)	Number of hospitalizations due to infections
Pasic et al. (26)	Observational study, prospective matched–control cohort (SCIg–IVIg)	Canada	Post–HSCT and HGG (IgG <700 mg/dL). *N* = 40 IVIg: *n* = 20, SCIg: *n* = 20 (prospective SCIg patients matched to concurrent IVIg patients)	SCIg: 0.1 g/kg/week IVIg: 0.4 g/kg every 28 days	6 months	Hospitalizations Consultations related to infections Costs ($Canadian) of drug, antimicrobial, hospitalizations, consultations, lab tests.
Vacca et al. (27)	RCT	Italy	MM and HGG (IgG <500 mg/dL). *N* = 46 SCIg: *n* = 24, No Ig: *n* = 22	SCIg 0.4–0.8 g/kg/month divided into four weekly infusions (frequency depended on IgG monthly levels) versus no Ig. Mean weekly SCIg: 0.08 g/kg	12 months (outcomes measurement)	Days of hospitalizations/year
Weeks et al. (28)	CUA	USA	CLL and HGG (IgG <50% LLN). Efficacy inputs derived from RCT *N* = 81	IVIg 0.4 g/kg/week versus no Ig	1–year time horizon	ICER at 1 year. Costs (1989 $US)
Windegger et al. (29)	CUA	Australia	HM and HGG (IgG threshold undefined). Efficacy inputs derived from patient cohort *N* = 13	IVIg 0.4 g/kg/month versus SCIg (dose NR) weekly	10–year time horizon	ICER. Costs (2018 $Australian)

Abbreviations: CAR, chimeric antigen receptor; CLL, chronic lymphocytic leukemia; CUA, cost-utility analysis; ED, emergency department; HGG, hypogammaglobulinemia; HM, hematological malignancies; HSCT, hematopoietic stem cell transplant; ICER, incremental cost-effectiveness ratio; IVIg, intravenous immunoglobulin; LLN, lower limit of normal; MM, multiple myeloma; NR, not reported; RCT, randomized controlled trial, SCIg, subcutaneous immunoglobulin.

### Resource use and costs in comparative studies of Ig

#### Before/after IVIg studies

Two observational studies using a before-and-after design (Table 2) reported hospitalizations due to infections (24;25). One study (25) compared patient outcomes 12 months before and after a low fixed dose of IVIg (10 *g* every 3 weeks) given to 15 patients with CLL and a history of recurrent infections. The median disease duration was 8.5 years, and most patients had advanced disease at the start of IVIg treatment. Results showed a significant (p = 0.047) reduction in hospitalizations due to infection following IVIg treatment. Of the 15 patients followed up, five discontinued IVIg.

**Table 2.** Resource use and costs in observational studies and RCT

References	*N*	Ig regimen	Comparison	Resource use and costs	Attrition *n* (%)	Follow up
Derman et al. (24)	274	IVIg 0.4 g/kg (flexible versus fixed intervals)	IVIg before/after stewardship program	ED or hospitalization due to infection, n (%) Prior autumn IVIg: 13 (27) Prior summer IVIg: 21 (24) After autumn IVIg: 13 (24) After autumn IVIg discontinued: 4 (9)	NR	3 months
Jurlander et al. (25)	15	IVIg 10 g/3wks	No IVIg versus After IVIg	Number of hospital admissions/year due to infection No IVIg = 16 vs/IVIg =5, p = 0.047	IVIg = 5 (33)^a^	12 months
Pasic et al. (26)	40	IVIg 0.4 g/kg/28 days SCIg 0.1 g/kg/wk	IVIg versus SCIg	Resource use IVIg versus SCIg Hospital days: 9 versus 41 Number of consultations related to infections: 0 vs 19 Cost per patient ($ Canadian) IVIg versus SCIg Ig plus administration (mean): $12,909 vs $8,833 Antimicrobials plus administration (mean): $191 versus $1,610 Hospitalizations (mean): $338 versus $1,538 Medical consultations (mean): $0 versus $144 Laboratory and diagnostic tests (mean): $42 vs $293 Total mean cost (95% CI): $13,480 (12,133–14,827) versus $12,418 (7,999–16,837) Total median cost (95% CI): $13,780 (9,908–21,561) versus $9,756 (645–40,734)	SCIg = 7 (33)^b^ IVIg = 0	6 months
Vacca et al. (27)	46	SCIg 0.4–0.8 g/kg/month	SCIg versus No Ig	Mean days of hospitalization/year due to severe infections SCIg = 8 vs No Ig = 121, p < 0.001	SCIg = 3 (11.5)^c^	12 months

Abbreviations: IVIg, intravenous immunoglobulin; NR, not reported; SCIg, subcutaneous immunoglobulin

a Reasons for discontinuation: fatal infection (*n* = 2), disease progression (*n* = 1), other disease (*n* = 1) and adverse event (*n* = 1).

b Reasons for discontinuation: intolerance (*n* = 3), noncompliance (*n* = 2), death due to transplant-related complications (*n* = 2).

c Reasons for discontinuation: adverse events (*n* = 3).

Another study (24), in the setting of either HSCT or chimeric antigen receptor T-cell therapy (CAR-T), retrospectively, assessed IVIg utilization and infection rates following the implementation of a pharmacy-led IVIg stewardship program aimed at reducing IVIg use in patients with hematological malignancies through more stringent access criteria and longer IVIg treatment intervals. Their key finding was that reducing IVIg use did not increase hospitalizations or emergency visits due to infection. This study reported cost-savings of US$44,700 by comparing the pre-program summer cohort with the post-program autumn cohort. However, the latter cohort had fewer patients, and these cost-savings were calculated as total costs of IVIg grams used, not costs per patient. In addition, seasonal differences in infection risk may have influenced these results. In order to account for seasonal variations in infections, the authors included additional data from 48 patients who received IVIg and 47 patients who discontinued IVIg in the previous autumn, but IVIg usage or costs were not presented for these two subgroups. Of the patients who discontinued IVIg, 83 percent reported the absence of severe infection in the previous period as the main reason for stopping treatment. There was no information on disease duration, stage, or line of treatment, and hematological diagnoses differed across the patient cohorts, with more patients with multiple myeloma in the pre-implementation cohorts.

#### SCIg versus No Ig

One RCT (27) compared SCIg to no Ig (nor prophylactic antibiotics) in 46 patients with multiple myeloma (Table 2). This study reported a significant (p < 0.001) annual reduction in hospitalization days/year due to severe infections in patients treated with SCIg compared to those not receiving Ig (mean days per year 8 versus 121). Overall, patient characteristics were balanced between the two groups; almost 30 percent had undergone prior HSCT, and over 50 percent of patients had received more than two lines of therapy. However, fewer patients in the SCIg group were treated with bortezomib-based therapies (50 versus 33.3 percent) and more were treated with immunomodulatory drugs (45.8 versus 31.8 percent). The mean SCIg treatment duration was 18 months, and none of the patients received prophylactic antibiotics.

#### IVIg versus SCIg

An observational study (Table 2) of 40 patients following HSCT reported resource use and cost per patient following 6 months of IVIg or SCIg (26). Twenty patients who started SCIg (14 of them transitioned from IVIg and 6 were de novo Ig) were age-matched to 20 patients receiving IVIg during the same 6-month period. Patients with SCIg attended more medical consultations due to infections and spent more days in the hospital than those receiving IVIg, but the total mean and median costs per patient were higher in the IVIg treatment group. This difference was mainly due to the higher IVIg cost per patient, including drug delivery costs. All patients treated with IVIg completed 6 months of treatment, whereas 25 percent of patients in the SCIg group discontinued SCIg due to adverse events or noncompliance, which may have decreased the effectiveness of SCIg. The authors noted that 30 percent of patients in the SCIg group were new to Ig, and this may have affected their findings, as more infections can occur at the beginning of Ig before sufficient Ig levels are reached. More patients in the SCIg group had acute leukemia, while myelodysplastic syndromes were more common in the IVIg group.

### Economic evaluations of Ig

We identified two economic evaluations of Ig (Table 3); one cost-utility analysis (CUA) of IVIg versus no Ig in CLL published in 1991 (28), and another CUA from 2019 comparing IVIg to home-based SCIg in patients with acquired HGG due to malignancies (hematological diagnosis not specified) (29). The first study suggested that IVIg was not cost-effective compared to no Ig, with a cost of US$6 million per quality-adjusted life-year (QALY) gained. The results from the CUA of IVIg versus SCIg suggested that SCIg was more cost-effective compared to IVIg, driven by lower incremental costs and higher incremental QALYs (i.e., SCIg was dominant).

**Table 3.** Cost-effectiveness results in economic evaluations

	Weeks et al. (28)	Windegger et al. (29)
Population	CLL patients	HM patients (diagnoses NR)^a^
Setting, currency	USA, 1989 US$	Australia, 2018 AUS$
Model type	Decision analysis	Markov cohort simulation
Perspective	Healthcare system^b^	Healthcare system
Time horizon	1 year	10 years
Comparison	IVIg 0.4/kg/3wks versus No Ig	IVIg 0.4 g/kg/month vs. SCIg (dose NR) weekly
Outcome selection	Transition probabilities from one RCT (*N* = 81) (30) Utility values obtained from 10 clinicians, who assigned values to each health state.	Transition probabilities Before/after observational cohort (*n* = 13). Utility values obtained from AQoL–6D administered to a larger cohort (*n* = 84). Both data sources were unpublished.
Ig cost	Cost/g = $30 Cost/infusion (incl. administration) = $910 Cost/person/year (incl. administration) = $15,470	Cost/g = $58.49 (domestic both IVIg/SCIg), $45.0 (imported IVIg), $57.43 (imported SCIg) Cost/person/week (excl. administration) = $357.29 (IVIg), $417.10 (SCIg)
Cumulative costs	NR	IVIg = $151,511
		SCIg = $144,296
Cumulative QALY	NR	IVIg = 3.07
		SCIg = 3.51
Incremental costs	IVIg – No Ig = $13,984	IVIg – SCIg = $7,215
Incremental QALY	IVIg – No Ig = 0.0023	IVIg – SCIg = −0.45
ICER	$6million/QALY	SCIg dominant
Sensitivity analysis	4.2–year time horizon, but results not reported	PSA

Abbreviations: CLL, chronic lymphocytic leukemia, HM, hematological malignancies, ICER, incremental cost-effectiveness ratio, IVIg, intravenous immunoglobulin, NR, not reported, PSA, probabilistic sensitivity analysis, QALY, quality-adjusted life-year, SCIg, subcutaneous immunoglobulin.

a Unpublished data, details from these patient cohorts not reported.

b The authors stated the model followed a societal perspective, but only direct medical costs were included.

The reporting of the economic evaluations had several gaps and generalizability to the current clinical landscape, and all patients with hematological malignancies may be limited (see Supplementary Table S2). The CUA of IVIg versus no Ig (28) was informed by an RCT of IVIg in 81 patients with CLL published in 1988 (30), and the costs applied to the model were derived from hospital costs in 1989 US$. The reporting of this economic evaluation was poor according to current standards (22), with key information missing with respect to model structure, time horizon, assumptions, and sensitivity analyses. The authors reported that a societal perspective was used, but only direct medical costs were included. The second CUA (29) used unpublished data from a cohort of 13 patients with acquired HGG secondary to malignancy or associated treatment who received IVIg and transitioned to SCIg after 12 months, but the study did not specify whether these were patients with hematological malignancies or which type. This study did not report which costs comprised the direct and indirect ward costs for treatment.

## Discussion

This systematic review highlighted key gaps in the literature regarding the costs and benefits of Ig therapy in hematological malignancies. Current economic evidence on Ig for the treatment of patients with hematological malignancies is scarce and the cost-effectiveness of Ig versus no Ig, or IVIg versus SCIg, remains highly uncertain.

Our search was designed to identify costing studies and economic evaluations of Ig, although citations were not restricted by study design and any study that reported cost or hospitalizations related to Ig use in the population of interest was included. Despite our wide inclusion criteria, only six relevant studies were identified, of which only two were economic evaluations (28;29). The remaining studies reported some hospitalization data in patients receiving Ig (24–27), and one of them compared per-patient costs of IVIg versus SCIg (26). The overall quality of the evidence was low and studies were highly heterogeneous, with different patient populations, interventions, and designs.

Only two economic evaluations were identified, and there was a high level of uncertainty around their results. The cost-effectiveness evaluation of IVIg versus no IVIg (28) has become outdated, with clinical inputs based on an RCT of patients with CLL published in 1988, utilities based on clinicians’ estimates, and unclear modeling assumptions and structure. The therapeutic landscape has vastly changed since 1988 with the introduction of targeted therapies, leading to increased survival but a higher incidence of HGG (1–3), which would impact on model estimates. The most recent economic evaluation (29) was based on a very small cohort of patients with undefined malignancies. This study did not apply different health state utilities to patients treated with IVIg and SCIg, despite indications of quality of life benefits in patients with primary and secondary immunodeficiencies treated with SCIg versus IVIg (31–33). We deduced that these patients had hematological malignancies and included this study in our review, given that a reference to the Australian criteria for Ig treatment in patients with HGG due to hematological malignancies was used to define secondary immunodeficiency, and hematologist consult fees were included in the model as specialist consultation. However, it was unclear whether patients with other malignancies were included. There were no data reported (or published elsewhere) on their disease duration, stage, treatment lines, or how transition probabilities were informed by infection rates. Utilities were derived from a patient survey including 84 patients, but patient characteristics were omitted.

The use of Ig has been increasing, but there were insufficient data on the total direct costs to the health system and indirect costs to the patient. High-quality evidence comparing the costs of IVIg versus SCIg in patients with hematological malignancies were lacking. The study by Pasic et al. (26) was the only one that compared mean costs per patient in the IVIg and SCIg cohorts, reporting lower total mean costs for SCIg than IVIg, which were driven by higher administration costs in the IVIg group. This study included a small number of patients who had undergone HSCT and may not be generalizable to the wider population of patients with hematological malignancies. Nevertheless, these results are consistent with cost savings associated with SCIg in patients with primary immunodeficiency disease (PID). A Canadian study suggested that transitioning patients with primary and secondary immunodeficiencies from IVIg to home-based SCIg had the potential to reduce nurse shortages and overall health care costs (34). Cost-savings following the transition from IVIg to SCIg were also estimated in economic evaluations of Ig replacement therapy in adult patients with PID, mainly due to reductions in hospital costs (35;36).

Only one study (24) in our review included patients receiving CAR-T therapy; these patients comprised only 8 percent of the total sample; therefore, no subgroup analyses were conducted. CAR-T therapy is associated with HGG, which is often profound and prolonged, and there is potential for an increase in Ig demand as CAR-T becomes more widely used. Nevertheless, the indication for prophylactic Ig treatment in patients with hematological malignancies receiving CAR-T remains controversial due to the lack of clinical and cost-effectiveness evidence (37;38). Guidance on the use of Ig in these patients is currently based on expert opinion, and careful stewardship of Ig treatment and individually tailored decision-making have been recommended (38).

The optimal use of Ig and its implications for the patient’s health, healthcare resources, and costs were uncertain, and in particularly, Ig use across patient subgroups, initiation, dosage, and treatment cessation remain unknown. Two of the studies identified in our review evaluated reduced IVIg dosage or intervals (24;25). Jurlander et al. (25) reported a reduction in hospitalizations in patients given a fixed low dose of IVIg compared to the previous period without Ig treatment, and Derman et al. (24) found that a stewardship program aimed at reducing IVIg use did not result in increased hospitalizations due to infections. Both studies were retrospective and had serious limitations. The first study (25) was published in 1994 and may not be sufficiently powered to detect treatment differences, given the very small number of patients and attrition. The latter (24) conducted a retrospective analysis of one institution’s program comprising different patient cohorts at various time points who may not have been comparable in their infection risk. In addition, the RCT by Vacca et al. (27) assessed serum IgG levels to adjust SCIg injection intervals, resulting in a lower weekly mean dose than the recommended 0.1 g/kg/week.

Criteria and guidelines for the use of Ig in secondary HGG vary worldwide; in Europe, severe or recurrent infections are a prerequisite for Ig treatment in patients with secondary HGG (39); in the United Kingdom, a trial of prophylactic antibiotics is required before Ig replacement (40), while in Australia, the presence of infections or prior trial of antibiotics is not required for patients with acquired secondary HGG to access government-funded Ig replacement therapy (41). In a Delphi exercise including European hemato-oncologists and immunologists (42), 63 percent agreed that IgG levels should be monitored in patients with hematological malignancies during routine visits, 73 percent agreed that the minimum Ig maintenance dose should be 0.4 g/kg body weight over a 3- to 4-week period, and 72 percent agreed that increasing the Ig dose should be considered in patients whose infections are not adequately controlled. International surveys of physicians prescribing Ig in secondary immunodeficiencies have also found variations in clinical practice; including Ig initiation and dosage, frequency of monitoring IgG levels to evaluate response, and treatment cessation (43;44). In France, a retrospective multicenter study in patients with secondary immunodeficiencies, not restricted to those with hematological malignancies, estimated that inappropriate use of Ig treatment can amount to more than 12 million euros, including the costs of hospital admissions (17). The total cost burden of Ig therapy and the impact of treatment variations in patients with hematological malignancies requires further research.

There are several limitations in the included evidence. Only studies published in English were included. It was not possible to conduct a meta-analysis due to the limited evidence and heterogeneity across the studies. There was a high degree of variation with respect to populations, interventions, study design and duration, and outcome reporting. Very limited cost data were provided, with only one study reporting costs per patient and two economic evaluations. Of the few studies that provided cost data, different currencies were used and the year of cost measurement varied widely (from 1989 to 2021). Two of the studies, including the only economic evaluation comparing IVIg to no Ig, were published in the 1990s before B-cell targeted therapies were introduced in current hematological practice. Most studies were observational in design and had a very small number of patients, which increased their risk of bias and limited their power to detect differences in hospitalizations due to infections.

### Future research

Given the current lack of data on the cost and cost-effectiveness of Ig in this population, further health economic research is urgently needed. We suggest several key areas:

- Research into the optimal use of Ig to clarify the most appropriate dosage, time of initiation and treatment cessation. Both clinical trials and registry data may help to identify which patients are more likely to benefit from Ig treatment, and thus avoid low value use.
- Real world data will enable the evaluation of variations in clinical practice and cost implications for the patient and the health system, including wider societal costs.
- Prospective studies are needed to assess long-term outcomes of Ig treatment, including quality of life measures to derive health care utilities, and the impact of novel therapies on Ig utilization.
- High-quality costing studies are required to better understand the total costs of Ig treatment, including both direct and indirect costs, and the economic impact of the transition from IVIg to SCIg.
- Considering potential improvements in quality of life of patients treated with SCIg, it will be important to assess whether home-based SCIg treatment translates into fewer hospitalizations and lower economic burden to the health system and the patient.
- Robust health economic models should be developed to understand the long-term benefits and costs of Ig treatment, as well as comparing IVIg versus SCIg.
- Future economic studies of Ig treatment should follow current reporting standards, such as CHEERS, so that good quality evidence may inform clinical decision-making.

## Conclusions

This review highlights the insufficient evidence on the cost and cost-effectiveness of Ig treatment in hematological malignancies, despite the increasing use of Ig in this population. The total costs associated with Ig treatment beyond product costs remain unknown, in particular costs associated with the administration of Ig and hospitalizations due to infections. As the use of B-cell targeted therapies for hematological malignancies increases, so does the likelihood of developing HGG, leading to higher use of Ig and associated costs. Understanding the cost-effectiveness of Ig is necessary to ensure a more efficient and equitable use of this limited resource and decrease the risk of Ig shortages. Addressing the identified knowledge gaps not only has the potential to result in major cost savings to health systems but will also inform current practice and improve patient outcomes.

## Supporting information

Carrillo de Albornoz et al. supplementary material Carrillo de Albornoz et al. supplementary material

## Supplementary material

The supplementary material for this article can be found at http://doi.org/10.1017/S026646232400028X.

## Other information

The protocol of this review was registered on Prospero: item CRD42022321908.
