# Supplementary material for: A systematic review of the cost and cost-effectiveness of immunoglobulin treatment in patients with hematological malignancies
Source: Int J Technol Assess Health Care. 2024 May 16;40(1):e32. doi: 10.1017/S026646232400028X (PMC11569901; doi:10.1017/S026646232400028X)
Supplement: Carrillo de Albornoz et al. supplementary material [file S026646232400028Xsup001.docx]

**Supplement to: “A systematic review of the cost and cost-effectiveness of immunoglobulin treatment in patients with hematological malignancies”**

Search strategy

The following databases were searched:

EBM Reviews - Cochrane Database of Systematic Reviews <2005 to March 24, 2022>

EBM Reviews - ACP Journal Club <1991 to March 2022>

EBM Reviews - Database of Abstracts of Reviews of Effects <1st Quarter 2016>

EBM Reviews - Cochrane Clinical Answers <March 2022>

EBM Reviews - Cochrane Central Register of Controlled Trials <January 2022>

EBM Reviews - Cochrane Methodology Register <3rd Quarter 2012>

EBM Reviews - Health Technology Assessment <4th Quarter 2016>

EBM Reviews - NHS Economic Evaluation Database <1st Quarter 2016>

Embase Classic+Embase <1947 to 2022 March 28>

Ovid MEDLINE(R) and Epub Ahead of Print, In-Process, In-Data-Review & Other Non-Indexed Citations, Daily and Versions <1946 to March 28, 2022>

**Table S1. Search strategy**

| 1 | exp h$ematologic neoplasm$/ | 2787195 |
| --- | --- | --- |
| 2 | (h$ematologic$ and (malignanc$ or neoplasm)).ti,ab. | 83842 |
| 3 | (myeloma or leuk$emia or lymphoma).mp. | 1509755 |
| 4 | exp lymphoma/ | 543567 |
| 5 | (lymphoma or lymphoproliferative or non-hodgkin).mp. | 682784 |
| 6 | ((chronic adj lymphocytic adj leuk$emia) or CLL).mp. | 66668 |
| 7 | exp multiple myeloma/ | 141761 |
| 8 | (myeloma or (plasma adj cell)).mp. | 252649 |
| 9 | or/1-8 | 3645597 |
| 10 | exp immunoglobulin/ | 1557983 |
| 11 | ((immunoglobulin or Ig or IgG) adj (infusion$ or replacement$)).mp. | 4176 |
| 12 | ((intravenous or intramuscular or subcutaneous) adj2 (immunoglobulin$ or antibod$ or gammaglobulin$ or Ig or IgG)).mp. | 53872 |
| 13 | (IVIG or SCIG).mp. | 31382 |
| 14 | or/10-13 | 1572271 |
| 15 | cost of illness/ | 52538 |
| 16 | Health Care Costs/ | 221372 |
| 17 | (expenditure? adj3 (health or direct or indirect)).mp. | 44529 |
| 18 | (out-of-pocket adj2 (payment? or expenditure? or cost? or spending or expense?)).tw. | 14846 |
| 19 | (cost? adj2 (illness or disease or sickness)).tw. | 13517 |
| 20 | (resource or budget or cost*).ti,ab. | 2110971 |
| 21 | Quality-adjusted life years/ | 50346 |
| 22 | QALY$.ti,ab. | 40484 |
| 23 | ((adjusted or quality-adjusted) adj2 year?).tw. | 73833 |
| 24 | (health adj2 utility).tw. | 7178 |
| 25 | ((utilit* or disutilit*) adj (score* or value)).tw. | 7307 |
| 26 | economic evaluation/ | 106648 |
| 27 | (cost* adj2 (effective* or utilit* or benefit* or minimi* or unit* or estimat* or variable*)).ti,ab. | 517020 |
| 28 | decision tree/ | 29873 |
| 29 | (econom* adj2 model*).ti,ab. | 15901 |
| 30 | (markov adj model).ti,ab. | 24203 |
| 31 | (decision* adj2 (tree* or analy* or model*)).ti,ab. | 74920 |
| 32 | or/15-31 | 2384437 |
| 33 | 9 and 14 and 32 | 4345 |
| 34 | (exp animal/ or animal.hw. or nonhuman/) not exp human/ | 12683339 |
| 35 | 33 not 34 | 3988 |
| 36 | limit 35 to english language | 3858 |
| 37 | remove duplicates from 36 | 3611 |

Quality assessment

**a) ROB2**
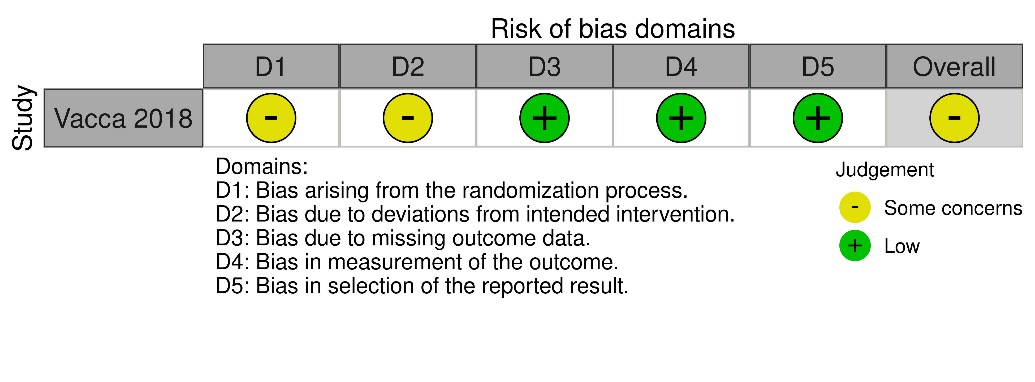


**b) ROBINS-I**


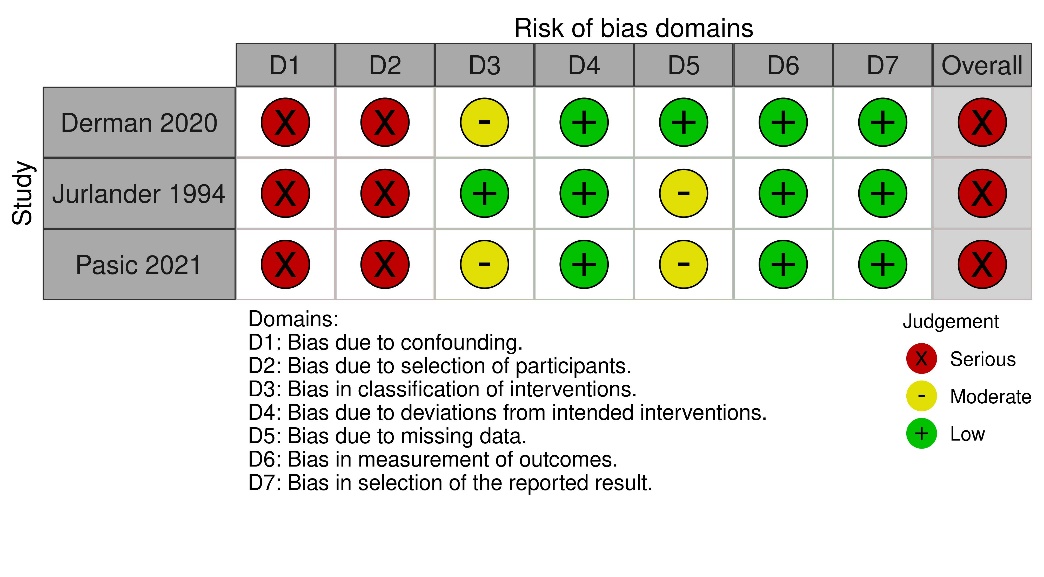


**Figure S1. Risk of Bias using a) ROB2 and b) ROBINS-I**

Risk of bias assessment using a) ROB2 for randomized trials , b) ROBINS-I for non-randomized studies

**Table S2. Quality assessment of Economic Evaluations using CHEERS 2022**

| **Items** | **Weeks 1991** | **Windegger 2019** |
| --- | --- | --- |
| Title | Cost effectiveness of prophylactic intravenous immune globulin in chronic lymphocytic leukemia (CLL) | Cost–utility analysis comparing hospital-based intravenous immunoglobulin (IVIg) with home-based subcutaneous immunoglobulin (SCIg) in patients with secondary immunodeficiency (SID) |
| Abstract | Context and methods: Data from a randomized controlled trial of 81 patients with CLL were used to develop a decision analysis model comparing the cost-effectiveness of intravenous immunoglobulin (IVIg) to no IVIg in patients with CLL over one year.  Results: IVIg resulted in a 0.8 QALY gain per patient per year at a cost of $6 million per QALY gained. IVIg led to a loss in QALY when the inconvenience of treatment was taken into account.  Conclusion: IVIg treatment might not result in improved quality or length of life and that it is extraordinarily expensive in comparison with other treatments. | Context and methods: Data from a cohort of 13 patients with SID attending a tertiary hospital were used to develop a Markov model to compare the cost-effectiveness of IVIg vs. SCIg from a healthcare system perspective, with a time horizon of 10 years.  Results: The cumulative cost for IVIg was A$151 511 and for SCIg A$144 296. The QALYs with IVIg were 307 and with SCIg 351. Based on the means, SCIg was the dominant strategy with better outcomes and at lower cost. The probabilistic  sensitivity analysis showed that 883% of the 50 000 iterations fell below the nominated willingness to pay threshold of A$50 000 per QALY.  Conclusion: the home-based SCIg treatment option provided better health outcomes and cost savings. |
| Background and objectives | Results from an RCT of IVIg vs no IVIg in patients with CLL showed those receiving IVIg had a significant reduction in bacterial infections. The aim of this study was to determine whether prophylactic IVIg led to an overall clinical benefit and to examine its cost-effectiveness compared to no IVIg. | Home-based SCIg was reported to be a cheaper and more efficient option compared to hospital-based IVIg for primary immunodeficiencies. This study evaluated the cost-effectiveness of IVIg vs. SCIg in patients with SID. |
| Health economic analysis plan | Not reported | Not reported |
| Study population | 81 CLL patients (RCT 1-year follow up). | 13 patients with HGG due to malignancy (diagnoses undefined* before/after design) |
| Setting/location | USA | Australia |
| Comparators | Ig 400mg/kg vs. No Ig | IVIg/4 wks vs. SCIg/wk |
| Perspective | Reported as societal. However, only direct costs were included, indicating a healthcare system perspective. | healthcare system |
| Time horizon | Unclear. 1 year in base case but 4.2 years in sensitivity analysis (not reported) | 10 years |
| Discount rate | 5% | 5% |
| Selection of outcomes | QALYs used to estimate benefits and harms | QALYs used to estimate benefits and harms |
| Measurement of outcomes | QALYs were calculated from utility values. Utilities derived from physicians (n=10) who assigned quality adjustments to each state | QALYs were calculated from utility values. Utility weights calculated from AQoL-6D in 84 patients with SID (characteristics of these patients NR) |
| Valuation of outcomes | Based on RCT (N=81) in patients with CLL. One-year infection rates used as annual probabilities. The overall 1-year mortality rate in the RCT was used to estimate the probability of death in both groups as the number of deaths was too small to detect between group differences. | Based on a cohort (N=13) of patients with HGG due to malignancy, before/after observational study (unpublished data); 1-year IVIg and 1-year SCIg. Infection outcomes were collected retrospectively from medical records and GPs. Modelled outcomes were incidence of infection and mortality. |
| Measurement and valuation of resources and costs | Cost of treatment, adverse reactions, and infections were derived from data from two Boston hospitals (USA). Administration costs were estimated for a duration of infusion of 2.5 hours, on the basis of an outpatient transfusion. It was not reported if these included consumables and hospital costs. | Cost of Ig product was obtained from the National Blood Authority (Australia). Costs of treatment and infections were obtained from the hospital utilization of the patient cohort, Sunshine Coast Hospital&Health Service (Australia). Administration costs included consumables, ward costs, and pumps and training for SCIg. |
| Currency, date | 1989 US$ | 2018 AUS$ |
| Rationale and description of model | Decision analysis model, no details as to what type. Health states and transitions poorly reported. Time horizon unclear, model cycle not reported. | Markov cohort simulation model with six health states: No infection, infection, bronchiectasis no infection, bronchiectasis with infection, bronchiectasis with chronic Pseudomona A. infection, dead. Time horizon 10 years, weekly cycles. |
| Analytics and assumptions | Poorly reported. Assumptions about infections over time or Ig treatment cessation were not reported. | Transition probabilities calculated from annual infection rates. Data on infection rates informing health state probabilities were not clearly reported. No data extrapolations were conducted. |
| Characterizing heterogeneity | NR | NR |
| Characterizing distributional effects | NR | NR |
| Characterizing uncertainty | Poorly reported. Model modified with a time horizon of 4.2 years but extrapolations or ICER NR. | PSA reported. |
| Patient engagement | NR | NR |
| Study parameters | All transition probabilities, utilities and costs were reported. Transition probabilities were based on published infection rates (Gale et al. 1988). Distributions were not reported. | All transition probabilities, utilities, and costs (plus distributions) were reported. However, rates of infections were not included or how transition probabilities were derived. |
| Main results | One-year ICER reported. | Ten-year ICER reported. |
| Effect of uncertainty | Poorly reported. ICER at 4.2 years NR. No other sensitivity analyses reported. | PSA reported. |
| Discussion, findings limitations, generalizability, current knowledge | Poor generalizability given changes in treatments and costs in the past 30 years. Life expectancy in CLL has also increased during this time, and the introduction of B-cell depleting therapies may have altered the incidence of infections | Poor generalizability given the small sample size clinical inputs are based on and lack of patient characteristics (e.g., diagnosis NR). Unclear if the 84-patient sample used to obtain utility values had hematological malignancies. Unclear what transition probabilities were based on, and how the probability of death was estimated for the different health states. Infection rates or mortality NR. No distinction between infections and severe infections (requiring hospitalization). Unclear how the ward costs were calculated. |
| Source of funding | Kellogg Program for Training in Research in Clinical Effectiveness | University of the Sunshine Coast, National Blood Authority, CSL Australia, the Wishlist Sunshine Coast Health Foundation. |
| Conflicts of interest | NR | Founders had no impact on study design, analysis, interpretation or writing. |
| Other comments | it was assumed that patients who had an infusion reaction would no receive further Ig and had the same infection rates as treated controls. | No discussion about discontinuation of Ig |

Abbreviations: CLL=chronic lymphocytic leukemia, HGG=hypogammaglobulinemia, ICER=incremental cost-effectiveness ratio, Ig=immunoglobulin, IVIg=intravenous immunoglobulin, NR=not reported, QALY=quality-adjusted life year, SCIg=subcutaneous immunoglobulin, SID=secondary immunodeficiency.

* The author was contacted to clarify if these were hematological malignancies, but the query was not answered.
